# Soft tissue response in orthognathic surgery patients treated by bimaxillary osteotomy: cephalometry compared with 2-D photogrammetry

**DOI:** 10.1007/s10006-012-0330-0

**Published:** 2012-05-05

**Authors:** Jan Rustemeyer, Alice Martin

**Affiliations:** Department of Oral and Maxillofacial Surgery, Medical Centre Bremen–Mitte, School of Medicine, University of Göttingen, Bremen, Germany

**Keywords:** Cephalometry, Photogrammetry, Orthognathic surgery, Soft tissue, Prediction

## Abstract

**Purpose:**

Since improvement of facial aesthetics after orthognathic surgery moves increasingly into the focus of patients, prediction of soft tissue response to hard tissue movement becomes essential for planning. The aim of this study was to assess the facial soft tissue response in skeletal class II and III patients undergoing orthognathic surgery and to compare the potentials of cephalometry and two-dimensional (2-D) photogrammetry for predicting soft tissue changes.

**Material and methods:**

Twenty-eight patients with class II relationship and 33 with class III underwent bimaxillary surgery. All subjects had available both a traced lateral cephalogram and a traced lateral photogram taken pre- and postsurgery in natural head position (median follow-up, 9.4 ± 0.6 months).

**Results:**

Facial convexity and lower lip length were highly correlated with hard tissue movements cephalometrically in class III patients and 2-D photogrammetrically in both classes. In comparison, cephalometric correlations for class II patients were weak. Correlations of hard and soft tissue movements between pre- and postoperative corresponding landmarks in horizontal and vertical planes were significant for cephalometry and 2-D photogrammetry. No significant difference was found between cephalometry and 2-D photogrammetry with respect to soft to hard tissue movement ratios.

**Conclusions:**

This study revealed that cephalometry is still a feasible standard for evaluating and predicting outcomes in routine orthognathic surgery cases. Accuracy could be enhanced with 2-D photogrammetry, especially in class II patients.

## Introduction

During recent decades, orthognathic surgery has become widely accepted as the preferred method of correcting moderate to severe skeletal deformities including facial aesthetics. Recognition of aesthetic factors and prediction of the final facial profile play an increasingly important role in orthognathic treatment planning, since the facial profile produced by orthognathic surgery is highly significant for patients [1–3]. Many studies have attempted to evaluate the relationship between hard tissue surgery and its effect on the overlying soft tissue for predicting facial changes [4–6]. Three-dimensional (3-D) imaging techniques, including computer tomography, video imaging, laser scanning, morphanalysis, 3-D sonography, and, recently, 3-D photogrammetry [7–13] have been developed to highlight the relationship between hard and soft tissue movements, but details of this relationship, particularly in the vertical direction, have varied and not been fully clarified [14]. However, the assessment of visible volume changes with an optical 3-D sensor can be carried out with considerable accuracy and provides the opportunity to complete the cephalometric analysis in cases of midfacial distractions and asymmetric craniofacial situations [15].

For routine orthognathic surgery cases, cephalometry and 2-D photogrammetry are common and less expensive tools that may have the potential to analyze and predict the resulting profile. However, it is remarkable that no recent report offers a comparison between both conventional methods of indirect anthropometry. Therefore, the objective of this study was to assess the facial soft tissue response in skeletal class II and III patients treated by bimaxillary orthognathic surgery both cephalometrically and with 2-D photogrammetry and to compare their ability to predict postoperative outcomes. Hence, the relevant questions were whether both methods have the capacity to complement one another or not and in which cases.

## Patients and methods

### Patients' sample

Twenty-eight patients who had undergone bimaxillary surgery for a class II relationship (mean age, 24.5 ± 4.9 years; 18 females and 10 males), and 33 patients who had undergone bimaxillary surgery for a class III relationship (mean age, 23.4 ± 3.7 years; 20 females and 13 males) were selected from adult treatment records. Bimaxillary surgery consisted of LeFort I osteotomy with maxillary advancement and/or impaction and bilateral sagittal split ramus osteotomy carried out for mandibular setback or advancement. Setback of the maxilla was not done. No additional surgical procedures were performed on the midface or chin, such as infraorbital augmentation, distraction, rhinoplasty, or genioplasty. Exclusion criteria to avoid any bias were patients' findings that exceeded routine orthognathic planning. These were patients with an anterior open bite of more than 1 cm, facial asymmetry with occlusal cants in the frontal plane, midline deviations and mandibular border asymmetry, matured cleft lip and palate, severe congenital facial deformity, and posttraumatic deformity.

All subjects had available both a lateral cephalogram and a lateral photogram in the natural head position (NHP) taken before orthodontic appliances were applied and 9 months postsurgery, after removal of the orthodontic appliances and osteosynthesis materials (median follow-up, 9.4 ± 0.6 months).

### Lateral cephalometry

Subjects were positioned in the cephalostat (Orthoceph; Siemens AG, Munich, Germany), and then the head holder was adjusted until the ear rods could be positioned into the ears without moving the patient. All radiographs were taken in the NHP with teeth together and lips in repose and with a metric ruler in front of the midfacial vertical line. No occipital supplement was used. According to cephalometric standards, the film distance to the X-ray tube was fixed at 150 cm, and the film distance to the midsagittal plane of the patient's head, at 18 cm.

**Fig. 1:**
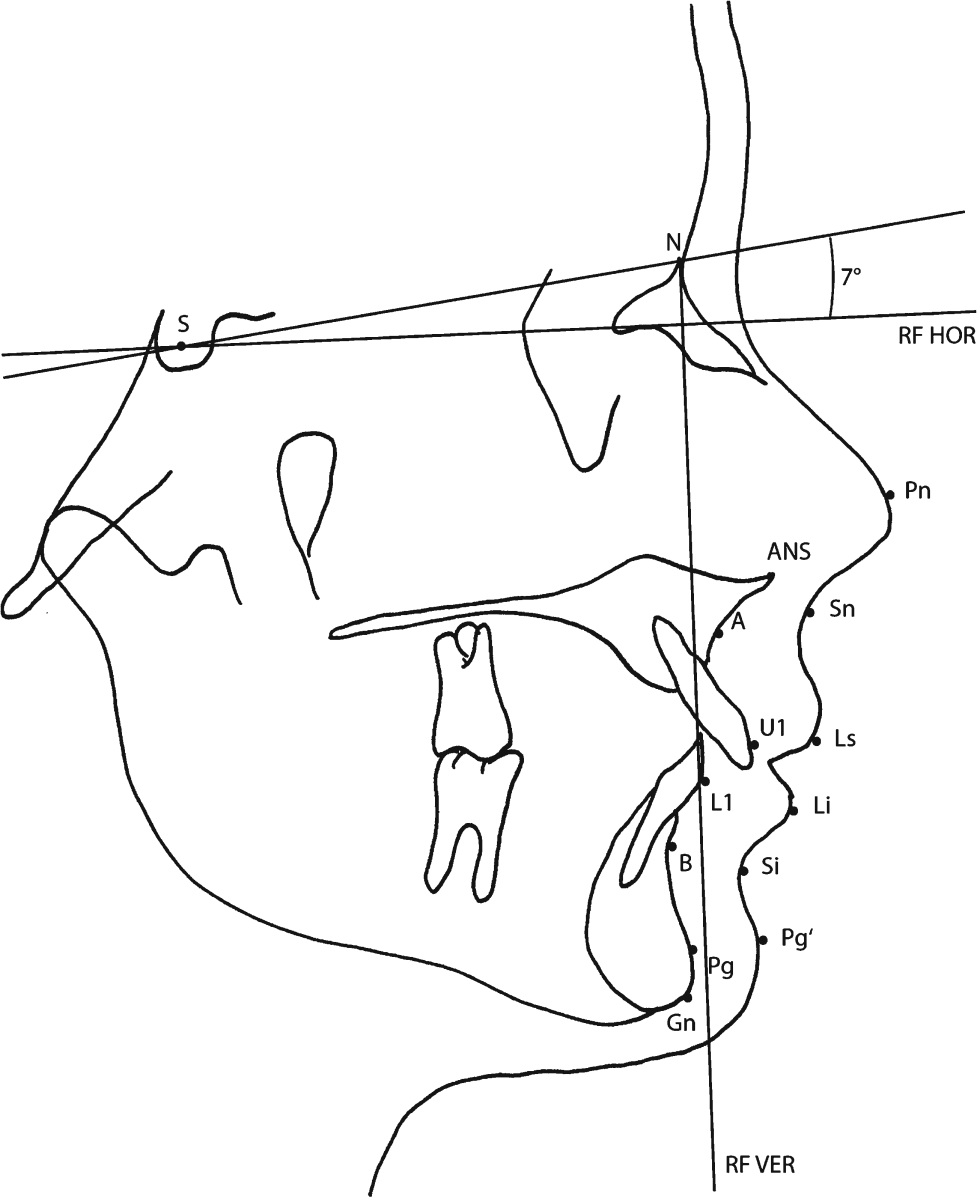
Hard and soft tissue landmarks and reference lines for tracing cephalograms. *N* nasion, *S* sella, *A* point A, *B* point B, *L1* lower incisor, *U1* upper incisor, *Gn* gnathion, *Pg* pogonion, *ANS* anterior nasal spine, *Pn* pronasale, *Sn* subnasale, *Ls* labrale superius, *Li* labrale inferius, *Si* labiomental sulcus, *Pg*′ soft tissue pogonion, *RF HOR* horizontal reference line, *RF VER* vertical reference line

**Fig. 2:**
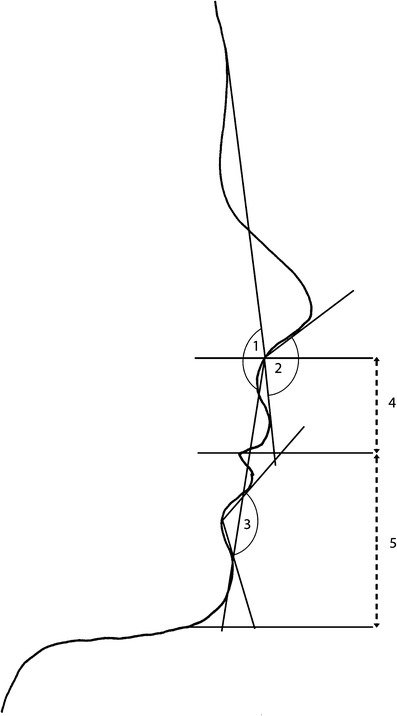
Soft tissue angles and distances for tracing cephalograms and photograms. *1* Facial convexity, *2* nasolabial angle, *3* labiomental angle, *4* upper lip length, *5* lower lip length

Tracings were done for all cephalograms. After loading the cephalogram into a PC, the ruler was used to size the cephalogram image in the software program (Adobe Photoshop version 7.0; Adobe Systems, San Jose, CA, USA), so that 1 mm on the rule represented 1 mm of actual scale (life-size) in the software program. The landmarks were identified manually by a single examiner using the photographic software. Soft and hard tissue landmarks of the cephalograms were traced using a modified version of the analysis of Legan and Burstone [16] and Lew et al. [17] (Figs. 1 and 2). Therefore, the horizontal reference line was constructed by raising a line 7 ° from sella-nasion, and a line perpendicular to this at nasion was used as the vertical reference line. Movement of hard and soft tissue landmarks from pre- to postsurgery was measured in millimeters to the horizontal and vertical reference lines. The corresponding angles were constructed and measured in degrees in the pre- and postsurgical cephalograms. Differences were recorded as the surgical change.

### 2-D photogrammetry

**Fig. 3:**
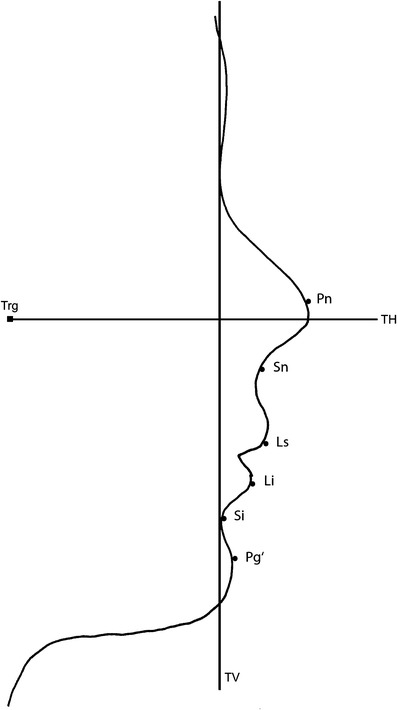
Soft tissue landmarks and reference lines for tracing photograms. *TV* true vertical, *TH* true horizontal, *Trg* tragus. Further abbreviations as given in Table 1

Subjects were asked to sit on a chair in front of a pale blue background, maintain a straight back, and look straight ahead with a relaxed facial expression and eyes fully open, lips gently closed, and not smiling. A neck holder was then adjusted to help the subjects fix their NHP. For reproducibility, a simple, indirect light source on the ceiling was used, consisting of four 60-W fluorescent tubes to eliminate undesirable shadows from the contours of the facial profile. The subjects' faces were photographed in right lateral view, together with a metric scaled ruler in front of the midfacial vertical line (true vertical (TV)). A high-resolution digital camera with a flash (Canon 450D; Tokyo, Japan) was firmly mounted on a photo stand 1 m in front of the subject. All photographs were taken at 2,048 × 1,536 pixels resolution and saved in JPEG file format. Images were stored on the PC's hard drive and then transferred into the photographic software program. The lateral photographs were adjusted to life-size according to the cephalogram adjustment as above. Soft tissue landmarks, distances, and angles were traced with the tools of the software. Additionally, TV on nasion and true horizontal (TH) (perpendicular to TV through the tragus) were constructed as reference lines for horizontal and vertical landmark movements. Pre- and postsurgical distances of each landmark toward reference lines were measured and differences were recorded as the vertical and horizontal surgical change, respectively, (Figs. 2 and 3).

### Statistics and reliability of measurements

The collected data were subjected to statistical analysis using the PASW statistical software package, version 18.0 (SPSS, Chicago, IL, USA). Differences between groups were evaluated using the paired *t* test. Results were considered significant if *p* < 0.05 and highly significant if *p* < 0.01. Pearson's correlation analysis was used to assess the degree of correlation between soft and hard tissue changes. The adjusted coefficient of determination (adj *R* ^2^) was used to assess the predictability of landmark movements (ranging from 0 = no prediction possible to 1 = accurate prediction possible).

Reliability of measurements was determined by randomly selecting ten cephalograms and ten lateral photograms to repeat the tracings by a second senior examiner. The method error was calculated using the formula: $\sqrt {{\sum {{({X_1} - {X_2})}^2}}} /2n$ in which *X*_1_ was the first measurement; *X*_2_, the second measurement; and *n*, the number of repeated records. All respective values of method error calculation for the linear measurements ranged between 0.32 and 0.48 mm for cephalometry and between 0.35 and 0.51 mm for 2-D photogrammetry and for angular measurements, between 1.4 and 5.2 ° and between 1.6 and 4.9 °, respectively. Significant differences between the reliability of photogrammetry and cephalometry could not be obtained.

## Results

### General findings

Significant differences between females and males could not be obtained cephalometrically or photogrammetrically, nor with respect to angular or distance measurements, pre- or postoperative, nor landmark movements. Therefore, gender was not considered further.

Hard tissue angles assessed by cephalometry changed significantly from pre- to postsurgery in class II and III patients (sella-nasion point A (SNA), *p*_class II_ = 0.041, *p*_class III_ = 0.015; sella-nasion point B (SNB), *p*_class II_ = 0.009, *p*_class III_ = 0.008; A intersect B (ANB), *p*_class II_ = 0.016, *p*_class III_ <0.001; nasion point A pogonion (NAPg), *p*_class II_ = 0.043, *p*_class III_ < 0.001).

### Soft tissue angles and distances

**Fig. 4:**
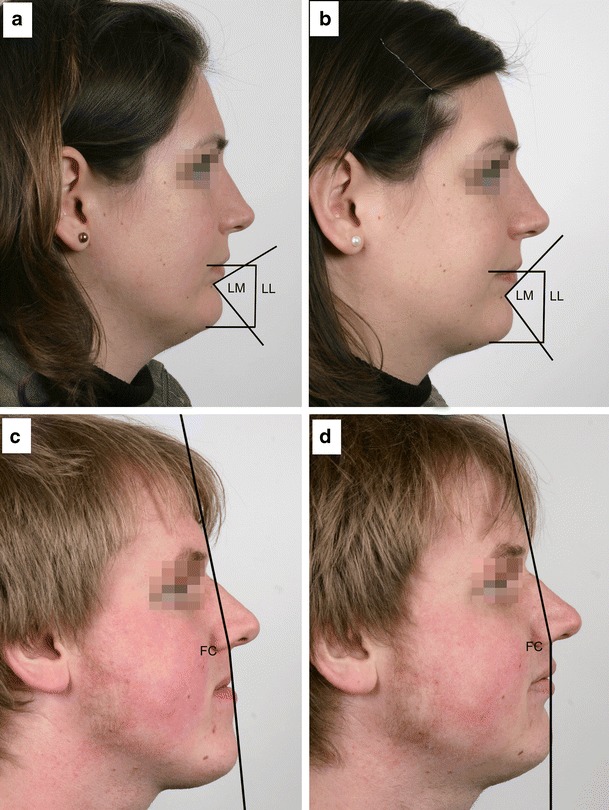
Screenshots of traced lateral photograms. Pre- to postsurgical changes of lower lip length (*LL*) and labiomental angle (*LM*) in class II patients (**a** presurgery and **b** postsurgery) and changes of facial convexity (*FC*) in class III patients (**c** presurgery and **d** postsurgery) revealed high significance

Significant differences between pre- and postsurgical measurements could be found for facial convexity, labiomental angle, and lower lip length by cephalometric and photogrammetric analyses (Table 1). Pre- to postsurgical changes of facial convexity in class III patients and changes of lower lip length and labiomental angle in class II patients revealed high significance (*p* < 0.01; Fig. 4). No significant changes from pre- to postsurgery could be found for the nasolabial angle or upper lip length.

**Table 1:** Pre- and postsurgical measurements of soft tissue angles and distances

		Photogrammetry			Cephalometry		
		Presurgery	Postsurgery		Presurgery	Postsurgery	
Parameter	Class	Mean ± SD	Mean ± SD	*p* value	Mean ± SD	Mean ± SD	*p* value
Facial convexity (°)	II	159.1 ± 4.8	165.9 ± 5.1	0.023*	159.8 ± 2.3	163.5 ± 3.4	0.015*
	III	178.8 ± 5.9	172.1 ± 6.1	<0.001**	178.8 ± 5.9	170.8 ± 7.3	<0.001**
Nasolabial angle (°)	II	111.2 ± 7.4	109.2 ± 9.2	0.671	111.4 ± 10.1	111.2 ± 7.5	0.976
	III	105.4 ± 12.4	104.6 ± 13.3	0.835	102.1 ± 14.2	103.2 ± 14.7	0.804
Labiomental angle (°)	II	119.1 ± 11.9	135.9 ± 9.8	0.013*	120.8 ± 7.4	134.2 ± 9.9	0.021*
	III	132.8 ± 14.6	121.1 ± 15.8	0.013*	127.4 ± 12.9	115.5 ± 13.8	0.004**
Upper lip length (mm)	II	13.5 ± 1.7	13.9 ± 1.3	0.621	13.9 ± 1.9	13.8 ± 1.9	0.533
	III	12.4 ± 1.6	13.1 ± 1.6	0.134	12.5 ± 2.1	13.1 ± 1.8	0.317
Lower lip length (mm)	II	24.7 ± 3.1	30.5 ± 3.3	0.006**	29.9 ± 2.3	29.9 ± 2.3	0.007**
	III	31.2 ± 3.4	28.8 ± 3.9	0.029*	31.6 ± 2.9	28.4 ± 2.7	0.003**

*p < 0.05; **p < 0.01; significant levels

### Soft tissue landmarks

The measurements of pre- to postsurgical soft tissue landmark movements did not differ significantly between photogrammetry and cephalometry (Table 2). In class III patients, the greatest movements were found photogrammetrically and cephalometrically for soft tissue pogonion (Pg′) in the horizontal and for labiomental sulcus (Si) in the vertical dimension. In class II patients, Si movements assessed by photogrammetry and Pg′ movements assessed by cephalometry revealed the greatest movements in both horizontal and vertical directions.

**Table 2:** Pre- to postsurgical movements of soft tissue landmarks in horizontal and vertical dimensions assessed by photogrammetry and cephalometry

		Photogrammetry	Cephalometry	
		Movement (mm)	Movement (mm)	*p* value
Parameter	Class	Mean ± SD	Mean ± SD	
Horizontal dimension				
Landmark				
Pn	II	0.9 ± 0.8	0.6 ± 0.5	0.251
	III	1.4 ± 2.6	1.1 ± 0.9	0.761
Sn	II	2.1 ± 0.8	2.2 ± 0.9	0.883
	III	2.4 ± 1.6	1.2 ± 3.1	0.784
Ls	II	2.5 ± 0.5	2.3 ± 1.7	0.831
	III	2.2 ± 1.6	1.1 ± 2.5	0.874
Li	II	2.5 ± 0.8	2.2 ± 1.3	0.441
	III	−3.2 ± 2.1	−4.8 ± 3.1	0.376
Si	II	2.7 ± 0.5	2.3 ± 0.8	0.421
	III	−5.4 ± 2.9	−5.9 ± 3.4	0.776
Pg′	II	2.5 ± 1.1	3.3 ± 1.2	0.232
	III	−6.8 ± 4.1	−6.1 ± 4.3	0.769
Vertical dimension				
Landmark				
Pn	II	0.1 ± 0.8	0.3 ± 0.5	0.451
	III	0.6 ± 1.1	0.4 ± 0.5	0.736
Sn	II	0.2 ± 0.9	−0.2 ± 0.7	0.525
	III	0.6 ± 0.4	0.2 ± 0.4	0.688
Ls	II	−0.5 ± 1.6	0.2 ± 0.9	0.418
	III	1.2 ± 0.8	1.4 ± 2.5	0.807
Li	II	−0.6 ± 0.8	0.3 ± 1.2	0.187
	III	1.2 ± 2.1	2.5 ± 2.6	0.411
Si	II	−1.3 ± 1.6	−0.2 ± 1.3	0.205
	III	1.8 ± 1.9	2.6 ± 1.9	0.283
Pg′	II	−1.2 ± 0.8	−0.7 ± 0.7	0.204
	III	1.4 ± 1.8	1.8 ± 2.3	0.199

### Correlations between soft and hard tissue changes

Significant correlations between soft and hard tissue changes (Table 3) occurred cephalometrically only in class III patients. Highly significant correlations were found between facial convexity and SNB, ANB, and NAPg and between lower lip length and SNB, ANB, and NAPg. Photogrammetrically significant correlations occurred in class II patients for labiomental angle and SNB, ANB, and NAPg, and in class III patients, for facial convexity and NAPg, for nasolabial angle and SNA, and for lower lip length and NAPg. Significant correlations for both class II and III patients could be shown between lower lip length and ANB.

**Table 3:** Significance of correlations between soft and hard tissue changes

Parameters^a^	Class	SNA	SNB	ANB	NAPg
Cephalometry					
Facial convexity	II	ns	ns	ns	ns
	III	ns	0.003**	<0.001**	<0.001**
Upper lip length	II	ns	ns	ns	ns
	III	ns	ns	0.032*	0.010*
Lower lip length	II	ns	ns	ns	ns
	III	ns	0.002**	<0.001**	0.003**
Photogrammetry					
Facial convexity	II	ns	ns	ns	ns
	III	ns	ns	ns	0.036*
Nasolabial angle	II	ns	ns	ns	ns
	III	0.034*	ns	ns	ns
Labiomental angle	II	ns	0.038*	0.037*	0.030*
	III	ns	ns	ns	ns
Lower lip length	II	ns	ns	0.027*	ns
	III	ns	ns	0.032*	0.047*

^a^Only parameters revealing at least one significance were considered

*ns* not significant

*p < 0.05; **p < 0.01; significant levels

Correlations of hard and soft tissue movements between pre- and postoperative corresponding landmarks in the horizontal and vertical planes revealed significance for both cephalometry and 2-D photogrammetry in class II and III patients (Table 4). Correlations could be found for both methods between subnasale (Sn) and point A (A), Si and point B (B), and Pg′ and pogonion (Pg) in the horizontal plane for class II and III patients. In the vertical plane for class II patients, correlations could be shown cephalometrically only for Sn and A, and photogrammetrically only for Pg′ and Pg. In class III patients, cephalometry and 2-D photogrammetry revealed both significant correlations between vertical movements of Sn and A, labrale superius (Ls) and upper incisor (U1), and Pg′ and Pg. In cases of significant correlation, adj *R* ^2^ was above the 0.7 level, representing a satisfactory accuracy for prediction.

**Table 4:** Significances between hard and soft tissue landmark movement correlations

Soft tissue parameter^a^	Hard tissue parameter^a^	Class	*p*_Sceph; H_	Adj *R* ^2^	*p*_Sphoto; H_	Adj *R* ^2^
Horizontal						
Sn	A	II	0.046*	0.717	0.011*	0.792
		III	0.044*	0.718	0.010*	0.891
Si	B	II	0.023*	0.707	0.038*	0.725
		III	0.034*	0.762	0.030*	0.778
Pg′	Pg	II	0.032*	0.752	0.015*	0.757
		III	0.010*	0.894	0.044*	0.720
Vertical						
Sn	A	II	0.036*	0.732	ns	0.121
		III	0.043*	0.721	0.016*	0.821
Ls	U1	II	ns	0.044	ns	0.044
		III	0.044*	0.721	0.018*	0.701
Pg′	Pg	II	ns	0.183	0.041*	0.712
		III	0.010*	0.889	0.030*	0.782

^a^Only parameters revealing at least one significance were considered

*p*_Sceph; H_ significance of correlation between cephalometrically assessed soft tissue landmark movement and corresponding hard tissue landmark movement, *p*_Sphoto; H_ significance of correlation between photogrammetrically assessed soft tissue landmark movement and corresponding hard tissue landmark movement, Adj *R* ^2^ adjusted coefficient of determination, *ns* not significant

*p < 0.05; significant level

### Soft-to-hard tissue movement ratios

Soft to hard tissue movement ratios in the horizontal and vertical planes for corresponding landmarks displayed a soft tissue response following hard tissue movement (Table 5). No significant difference could be obtained between cephalometry and 2-D photogrammetry with respect to the soft to hard tissue movement ratios.

**Table 5:** Soft to hard tissue movement ratios in horizontal and vertical dimensions for corresponding landmarks

Soft tissue parameter (S)	Hard tissue parameter (H)	Class	Ratio S (ceph) to H	Ratio S (photo) to H
Horizontal				
Pn	ANS	II	0.33	0.73
		III	0.25	0.35
Sn	A	II	1.83	1.73
		III	0.39	0.59
Ls	U1	II	1.11	1.76
		III	0.27	0.60
Li	L1	II	0.88	1.09
		III	0.03	0.56
Si	B	II	1.27	1.35
		III	1.20	1.13
Pg′	Pg	II	1.13	1.09
		III	0.98	1.15
Vertical				
Pn	ANS	II	0.33	0.33
		III	0.40	0.60
Sn	A	II	0.06	0.03
		III	0.20	0.80
Ls	U1	II	0.25	0.35
		III	0.60	0.80
Li	L1	II	0.25	0.15
		III	0.33	0.07
Si	B	II	0.25	0.37
		III	1.37	0.83
Pg′	Pg	II	0.33	0.57
		III	1.49	0.57

## Discussion

The results of this study showed that maxillary and mandibular movements with bimaxillary osteotomy were effective on soft tissues both in vertical and horizontal directions, and they improved facial convexity to approximate aesthetic norms. Arnett and Bergman [18, 19] described the facial profile according to the angle of facial convexity in class I (165–175 °), class II (<165 °), and class III profiles (>175 °). Following this classification, in our study, postsurgical class I facial convexity was achieved in class II and III patients and assessed by 2-D photogrammetry as well as by cephalometry. However, cephalometric and photogrammetric changes of the labiomental angle could be obtained only in class II patients. Fernández-Riveiro et al. [20] found that the labiomental angle should be evaluated with caution because of its high method error and variability. In this study as well, photogrammetrically and cephalometrically defined labiomental angle measurements revealed the highest variability of all measurements.

Whereas horizontal movement of soft tissue landmarks in class II and III patients—with the exception of labrale superius and inferius—was strongly correlated cephalometrically and 2-D photogrammetrically with hard tissue landmark movements, vertical movements of landmarks were mostly hard to predict. One reason might be that vertical mandibular movements, in our patients, were only minimal and beneath the capability of cephalometric and 2-D photogrammetric analyses, since patients with massive vertical deficits were excluded to avoid any bias in this study. Accordingly, Lin and Kerr [21] also found in their cohort that these may account for the increased difficulty in accurately predicting a change in the vertical dimension. In comparison, in the study of Nkenke et al. [15] using optical 3-D images for analyzing soft tissue advancement in patients undergoing midfacial distraction at 6 and 24 months postsurgically, means of vertical advancement of Sn (1.0 ± 1.0 and 0.4 ± 0.9 mm, respectively) and labrale superius (0.4 ± 1.1 and −0.2 ± 0.5 mm, respectively) were within the scope of the data assessed in this study by 2-D photogrammetry and cephalometry for class II and III patients. Hence, adequate accuracy of determination of vertical movements could be achieved with both methods in this study, and referring to the study of Nkenke et al. [15], the level of validity is acceptable. However, further studies are warranted to evaluate the concept of vertical changes in patients with extensive vertical discrepancies.

Findings in this study suggest that cephalometric and 2-D photogrammetric analyses complement one another in predicting soft tissue changes in orthodontic surgery patients. For the combination of both methods, at least one parameter for the maxilla (Sn–A) and one for the mandible (Pg′–Pg) became predictable for the vertical dimension with an acceptable adjusted coefficient of determination. Special attention should be given to soft tissue changes in class II patients, which cephalometrically revealed no significant correlation with hard tissue angular changes, whereas correlations could be obtained with 2-D photogrammetry. We therefore recommend supplementary 2-D photogrammetry for evaluating soft to hard tissue changes and cephalometric prediction, especially in class II patients.

Previous cephalometric findings have shown mandibular skeletal movement for the soft tissue chin at a ratio of between 0.9:1 and 1:1 [22, 23]. The results of this study support these historical observations cephalometrically as well as 2-D-photogrammetrically for class II and III patients. However, the labrale inferius in our study responded at a ratio of 0.88:1 cephalometrically and 1.09:1 photogrammetrically to the corresponding hard tissue movements in the horizontal plane in class II patients, but only at ratios of 0.03:1 and 0.56:1 in class III patients, respectively. This is cephalometrically much lower than the ratio found in other investigations in class III patients, which ranged from 0.6:1 to 0.75:1 [22, 23]. In comparison, with 2-D photogrammetry, the lower border of this range was nearly reached.

Standard error calculation suggests that the standards presented in this study for cephalometry and 2-D photogrammetry setups are ready for routine evaluation of soft tissue changes after orthognathic surgery. However, all ratios presented in this study and in the literature suggest that even a mathematically accurate prediction may involve bias [24]. This means that prediction and soft to hard tissue movement ratios must be evaluated on an individual basis and that they depend at least partly on the experience of the surgeon in his or her hand-setting of the maxilla during bimaxillary surgery. Furthermore, various types of operations—as well as the morphology of the anatomic structures—must be considered in predicting the outcome of facial surgery [25]. In comparison to data reported in another study from Nkenke et al. [26] using pre- and postsurgical 3-D facial surface images in patients undergoing LeFort I osteotomy, advancements of Sn and Ls were within the range of the results obtained in this study for horizontal movements of these parameters assessed with cephalometry and 2-D photogrammetry. Furthermore, the ratio of advancement between labrale superius and incision superius reported by Nkenke et al. [26] was 80 ± 94 % and comparable with our findings. In accordance to the ratios of vertical advancement and referring to the method of Nkenke et al. [26] again, validity of at least this ratio of horizontal advancement is adequate in our study. However, the 3-D facial surface images analysis possesses, moreover, the ability to predict volume increases or decreases especially in the malar midface region and could therefore improve the predictability of aesthetic soft tissue results. Future studies may reveal which orthognathic surgery cases are best suited for 3-D imaging techniques. The data of this study might be helpful.

## Conclusion

This study revealed that cephalometry and 2-D photogrammetry provide the option to complement one another to enhance accuracy in predicting soft tissue changes in orthodontic surgery, especially in class II patients.
